# Effect of Radiofrequency Ablation with Interventional Therapy of Hepatic Artery on the Recurrence of Primary Liver Cancer and the Analysis of Influencing Factors

**DOI:** 10.1155/2021/3392433

**Published:** 2021-10-13

**Authors:** Sishuo Zhang, Ge Zhao, Honglin Dong

**Affiliations:** ^1^Department of Surgery, Second Hospital of Shanxi Medical University, Taiyuan 030001, Shanxi Province, China; ^2^Department of General Surgery, Second Hospital of Shanxi Medical University, Taiyuan 030001, Shanxi Province, China; ^3^Department of Vascular Surgery, Second Hospital of Shanxi Medical University, Taiyuan 030001, Shanxi Province, China

## Abstract

**Background:**

The probability of liver cancer recurring in patients after surgery is a serious threat to liver cancer patients. Radiofrequency ablation is widely employed in liver cancer cases. We explored the therapeutic effects and influencing factors of radiofrequency ablation combined with hepatic artery intervention in patients with recurrence of primary liver cancer surgery.

**Methods:**

90 patients with primary liver cancer postoperative recurrence admitted to our hospital from January 2014 to February 2017 were selected as the research objects. The patients were randomly divided into the control group (*n* = 45) and combined treatment group (*n* = 45). The combined treatment group received radiofrequency ablation combined with hepatic artery interventional therapy, and the control group received hepatic artery interventional therapy. The short-term efficacy, AFP levels before and after treatment, and long-term survival results of the two groups were compared. Single-factor and multifactor analyses of the clinical information of the combined treatment group were carried out to find out the factors affecting the therapeutic effect of radiofrequency ablation combined with hepatic artery intervention on patients with recurrence of primary liver cancer.

**Results:**

The total effective rate of short-term curative effect of the combined treatment group was higher than the control group, and there was a statistically significant difference existing (*P* < 0.05). After treatment, two groups of patients' AFP levels were greatly lower than before treatment, the AFP levels of the combined treatment group were significantly lower than the control group, and there was a statistically significant difference (*P* < 0.05). The survival rates of patients in the combined treatment group at the sixth month, the first year, and the second year after treatment were significantly higher than those of the control group, and there was a statistically significant difference (*P* < 0.05). The univariate results showed that, in the combined treatment group, there were statistically significant differences between the effective group and the ineffective group in tumor diameter, intact capsule, liver cirrhosis, intrahepatic spread, and tumor adjacent to large blood vessels (*P* < 0.05). The outcomes of multivariate analysis indicated that tumor diameter ≥ 3 cm, incomplete capsule, intrahepatic spread, and tumor adjacent to large blood vessels were risk factors for ineffective recurrence of patients with primary liver cancer after radiofrequency ablation combined with hepatic artery intervention (*P* < 0.05). *Discussion*. Tumor diameter ≥ 3 cm, incomplete capsule, intrahepatic spread, and tumor adjacent to large blood vessels are risk factors for the ineffectiveness of radiofrequency ablation combined with hepatic artery interventional therapy for patients with recurrence of primary liver cancer. It is necessary to increase the range of radiofrequency treatment, increase the temperature of the radiofrequency needle, and strengthen postoperative follow-up interventions based on the specific conditions of the patient's tumor.

## 1. Introduction

Primary liver cancer refers to malignant liver tumors that originate from liver epithelial or mesenchymal tissues. It has the characteristics of high morbidity and high mortality. The clinical manifestations of its patients are pain, fatigue, and jaundice in the liver area [1]. Many patients will have liver cancer recurrence after treatment due to tumor metastasis, tumor cell residues, and other reasons. Statistics show that the probability of liver cancer recurring in patients within 5 years after surgery is more than 70%, which seriously threatens the long-term postoperative life of liver cancer patients. At present, the clinical treatments commonly used in the therapy of patients with recurrence of liver cancer include radiofrequency ablation and hepatic artery interventional chemotherapy [2, 3]. As thermal ablation therapy, radiofrequency ablation can use its local energy to kill tumor cells and has the advantages of less damage and fewer complications [4]. Radiofrequency ablation is widely applied in the therapy of liver cancer patients. However, there are few clinical reports about the therapeutic effect and influencing factors of radiofrequency ablation combined with hepatic artery intervention in patients with recurrence of primary liver cancer. This study retrospectively analyzed the clinical data and follow-up data of 90 patients with postoperative recurrence of primary liver cancer, observed the therapeutic effect of radiofrequency ablation combined with hepatic artery intervention on patients with postoperative recurrence of primary liver cancer, and analyzed its influencing factors. The research results are reported as follows.

## 2. Methods

### 2.1. Normal Information

Ninety patients with postoperative recurrence of primary liver cancer admitted to the Second Hospital of Shanxi Medical University, Taiyuan, Shanxi Province, China, from January 2014 to February 2017 were selected as the research objects. Inclusion criteria were as follows: (1) patients diagnosed with recurrence of primary liver cancer, (2) patients without mental disorders, (3) complete clinical data, and (4) signed informed consent. Exclusion criteria were as follows: (1) patients who have received immunotherapy, (2) patients with extrahepatic metastasis, and (3) patients who do not cooperate with follow-up. This study has been approved by the ethics committee of the Second Hospital of Shanxi Medical University, Taiyuan, Shanxi Province, China.

### 2.2. Clinical Data Collection

Clinical data of patients, including age, gender, tumor diameter, tumor number, tumor differentiation degree, complete capsule, liver cirrhosis, operation time, intrahepatic spread, pathological type, and tumor adjacent large blood vessels, were collected.

### 2.3. Radiofrequency Ablation Treatment Methods

Put the patient in the supine position, first locate the tumor, mark the puncture point under CT or B ultrasound, then use lidocaine at a concentration of 0.5% to give the patient local anesthesia, and finally give the patient a radiofrequency ablation treatment [5]. The parameters of the radiofrequency ablation machine are set as follows: the power range is 30–50 W and the temperature range is 90–105°C. When the patient's tumor diameter is less than 3 cm, radiofrequency ablation should be performed after a single-needle single-point puncture, and the ablation time is 8–10 minutes; when the patient's tumor diameter is greater than or equal to 3 cm, multistage multineedle single radiofrequency ablation should be used. The ablation time is 15–20 min [6].

### 2.4. Hepatic Artery Interventional Therapy

First, the Seldinger technique is used for intubation, and then the patient's celiac trunk arteriography or proper hepatic arteriography is performed to clarify the blood supply arteries of the patient's tumor. Then, under the guidance of DSA, the microcatheter is superselectively pushed into the tumor target vessel to infuse lobaplatin chemotherapeutics, and then a mixture of epirubicin and lipiodol emulsion embolism is injected.

### 2.5. Observation Index

The clinical efficacy of the two groups of patients was compared, including the following: (1) short-term efficacy: the tumor size was measured one month before and one month after treatment, and the modified solid tumor efficacy evaluation standard (mRE-CIST) [7, 8] was used to evaluate the efficacy of the patient; efficacy is divided into CR (complete remission: disappearance of all target lesions), PR (partial remission: reduction of the total length of the baseline lesions by at least 30%), SD (stable disease: reduction of the total length of the baseline lesions by less than 30% or the total length diameter increases but the increase is less than 20%), and PD (disease progression: the appearance of new lesions or the total length diameter of the baseline lesions increases by at least 20%); and the total effective rate = (CR + PR) number of cases/total cases × 100%; (2) alpha-fetoprotein (AFP) levels before and after treatment; (3) long-term survival results: the survival rate of patients at the 6th month, 1st year, and 2nd year after treatment. Based on the evaluation results of the solid tumors of the patients, 40 patients in the combined treatment group with complete remission and partial remission were included in the effective group, and 5 patients with disease progression and stable disease in the combined treatment group were included in the ineffective group.

### 2.6. Statistical Method

Data statistics are carried out using SPSS 23.0 software. Qualitative data are represented by *n* (%), and the *χ*^2^ test is used for comparison. Quantitative data are represented by (*x* ± *s*), and the *t*-test is used for comparison. Univariate analysis and logistic regression analysis were conducted to determine the factors affecting the therapeutic effect of radiofrequency ablation combined with hepatic artery intervention on patients with recurrence of primary liver cancer after surgery. *P* < 0.05 indicated statistical differences in the data.

## 3. Results

### 3.1. Comparison of the Short-Term Efficacy of the Two Groups of Patients

The total effective rate of combined treatment group was higher than that of control group, and there was a statistically significant difference (*P* < 0.05; Table 1).

### 3.2. Comparison of AFP Levels and Long-Term Survival Results between the Two Groups of Patients

No significant difference existed in the AFP levels of the two groups of patients before treatment (*P* > 0.05); the AFP levels of the two groups of patients after treatment were significantly lower than before treatment, and the AFP levels of the combined treatment group were greatly lower than those of the control group. There was a statistically significant difference (*P* < 0.05; Table 2).

### 3.3. Single-Factor Analysis of the Therapeutic Effect of Radiofrequency Ablation Combined with Hepatic Artery Intervention on Patients with Recurrence of Primary Liver Cancer

Univariate results indicated that no significant difference existed in age, gender, number of tumors, degree of tumor differentiation, intraoperative blood loss, operation time, hepatic port occlusion time, surgical margins, and pathological types between two groups of patients (*P* > 0.05). A statistically significant difference existed between the two groups of patients in tumor diameter, intact capsule, liver cirrhosis, intrahepatic spread, and tumor adjacent to large blood vessels (*P* < 0.05), as shown in Table 3.

### 3.4. Multifactor Analysis of the Therapeutic Effect of Radiofrequency Ablation Combined with Hepatic Artery Intervention on Patients with Recurrence of Primary Liver Cancer

In univariate analysis, statistically significant influencing factors (tumor diameter, complete capsule, liver cirrhosis, intrahepatic spread, and tumor adjacent to large blood vessels) were used as independent variables, and whether the patient was effective after treatment (effective = 0 and ineffective = 1) is the dependent variable, and the assignment is shown in Table 4. The results of multivariate analysis showed that tumor diameter ≥ 3 cm, incomplete capsule, intrahepatic spread, and tumor adjacent to large blood vessels were risk factors for ineffective recurrence of patients with primary liver cancer after radiofrequency ablation combined with hepatic artery intervention (*P* < 0.05; Table 5).

## 4. Discussion

The results of this research show that the total effective rate of radiofrequency ablation combined with hepatic artery intervention in patients with recurrence of primary liver cancer after surgery is higher than that of patients with hepatic artery intervention alone. After the treatment, the AFP level of all patients decreased, and the AFP level of the patients who used the radiofrequency ablation combined with hepatic artery intervention method decreased more compared with the patients who used the hepatic artery intervention alone. The survival rates of patients who received radiofrequency ablation combined with hepatic artery interventional therapy at the sixth month, the first year, and the second year were greatly higher than those of patients who received hepatic artery interventional therapy alone. It shows that the therapeutic effect of radiofrequency ablation combined with hepatic artery interventional therapy of patients with recurrence of primary liver cancer after surgery is better.

This study found that tumor diameter ≥ 3 cm, incomplete capsule, intrahepatic spread, and tumor adjacent to large blood vessels were risk factors for ineffective recurrence of patients with primary liver cancer after radiofrequency ablation combined with hepatic artery intervention (*P* < 0.05). The reasons may be as follows: (1) tumor diameter ≥ 3 cm is a risk factor for the ineffectiveness of radiofrequency ablation combined with hepatic artery intervention in patients with recurrence of primary liver cancer after surgery. The larger the diameter of the tumor, the faster the growth of tumor cells and the more likely it is to cause the rupture of the envelope [9], causing the tumor cells to spread to the surrounding tissues beyond the radiofrequency range, causing some tumor cells to fail [10]. Therefore, the efficacy of radiofrequency ablation combined with hepatic artery interventional therapy for patients with recurrent primary liver cancer is not good. Therefore, for patients with larger tumor diameters, the radiofrequency treatment range set by medical staff can be expanded based on the range of the lesion to ensure that all lesions and tumor cells that may be spread in the surrounding tissues are all killed, thereby improving patient efficacy [11, 12]. (2) Incomplete capsule is a risk factor for the failure of radiofrequency ablation combined with hepatic artery intervention in the treatment of patients with recurrence of primary liver cancer. The envelope of liver cancer refers to the extracellular matrix produced by avascular necrosis of the surrounding tissues due to the growth of the tumor [13, 14]. Studies have shown that [15] when the diameter of a patient's tumor is greater than 5 cm, the capsule is a protective factor for the patient's therapeutic effect. Patients with nonenveloped tumors have a shorter postoperative tumor-free survival period than patients with enveloped tumors. In patients with no tumor envelope, the tumor cells not only easily infiltrate the surrounding blood vessels but also may directly infiltrate the liver. In addition, it will increase the number of microlesions around the tumor [16, 17], resulting in patients without tumor envelope receiving treatment. Sometimes the treatment may be incomplete; there will be residual tumor cells or lesions that have not been cleaned, resulting in poor treatment effects for the patient. Therefore, medical staff should pay more attention to the therapy of patients with noncapsular tumors. After treatment, they should strengthen the follow-up of patients and pay close attention to their prognosis. Once residual lesions are found, they should be treated again in time. (3) Intrahepatic diffusion is a risk factor for the ineffectiveness of radiofrequency ablation combined with hepatic artery intervention in the treatment of patients with recurrence of primary liver cancer. The diffusion and metastasis of intrahepatic tumor in patients may lead to the failure of the lesion to be cleared during the treatment process and the infiltration of intrahepatic blood vessels by HCC cells [13], resulting in poor efficacy of radiofrequency ablation combined with hepatic artery intervention in the treatment of patients with postoperative recurrence of primary liver cancer. Therefore, when locating the tumor location of a patient, medical staff should carefully observe whether the patient has the possibility of intrahepatic metastasis of liver cancer cells [18]. For patients with intrahepatic metastasis of liver cancer cells, medical staff should take effective measures to intervene in time to prevent further diffusion. (4) Tumor adjacent to large blood vessels is a risk factor for the ineffectiveness of radiofrequency ablation combined with hepatic artery intervention in patients with recurrence of primary liver cancer after surgery. When a patient's liver cancer tumor is adjacent to large blood vessels, the temperature around the tumor may decrease due to the flow of blood vessels, thus affecting the effect of radiofrequency ablation and making the ablation incomplete [19]. As a result, the efficacy of radiofrequency ablation combined with hepatic artery intervention in the treatment of patients with recurrent primary liver cancer is not good. On the other hand, the proximity of large blood vessels to the tumor can change the shape of the ablation site, thus affecting the treatment outcome of patients [20, 21]. Therefore, for patients with tumors adjacent to large blood vessels, medical staff should consider increasing the ablation range and increasing the temperature of the radiofrequency needle to improve the treatment effect of the patient [22, 23].

## 5. Conclusions

In summary, tumor diameter ≥ 3 cm, incomplete capsule, intrahepatic spread, and tumor adjacent to large blood vessels are risk factors for poor therapeutic effect of radiofrequency ablation combined with hepatic artery intervention in patients with recurrent primary liver cancer. It is necessary to increase the range of radiofrequency treatment, increase the temperature of the radiofrequency needle, and strengthen postoperative follow-up interventions based on the specific conditions of the patient's tumor.

## Figures and Tables

**Table 1:** Comparison of the short-term efficacy of the two groups of patients (*n* (%)).

Group	*n*	CR	PR	NC	PD	Total effective rate
Control group	45	20 (44.44)	12 (26.67)	9 (20.0)	4 (8.89)	32 (71.11)
Combined treatment group	45	24 (53.33)	16 (35.56)	4 (8.89)	1 (2.22)	40 (88.89)
_*χ*_2						4.444
*P*						0.035

**Table 2:** Comparison of AFP levels and long-term survival results of the two groups of patients (*n* (%)).

Group	*n*	AFP (g/L)		Long-term survival outcome		
		Before treatment	After treatment	6 months	1 year	2 years
Control group	45	884.78 ± 78.38	423.64 ± 76.64	39 (86.67)	32 (71.11)	28 (62.22)
Combined treatment group	45	887.36 ± 76.64	218.64 ± 61.47	45 (100.0)	40 (88.89)	37 (82.22)
*t*/*χ*^2^		0.158	14.003	4.464	4.444	4.486
*P*		0.875	<0.001	0.026	0.035	0.034

**Table 3:** Single-factor analysis of the therapeutic effect of radiofrequency ablation combined with hepatic artery intervention on patients with recurrence of primary liver cancer after surgery (*n* (%)).

Factor	Effective group (*n* = 40)	Invalid group (*n* = 5)	*t*/*χ*^2^	*P*
*Age (years)*			0.003	0.958
≥60	21 (91.30)	2 (8.70)		
<60	19 (86.36)	3 (13.64)		

*Gender*			0.025	0.874
Male	22 (91.67)	2 (8.33)		
Female	18 (85.71)	3 (14.29)		

*Tumor diameter (cm)*			5.401	0.020
≥3	8 (66.67)	4 (33.33)		
<3	32 (96.97)	1 (3.03)		

*Number of tumors (n)*			0.070	0.791
≥2	17 (85.0)	3 (15.0)		
<2	23 (92.0)	2 (8.0)		

*Tumor differentiation*			0.703	0.402
I, II grade	28 (93.33)	2 (6.67)		
III, IV grade	12 (80.0)	3 (20.0)		

*Envelope intact*			6.321	0.012
Yes	33 (97.06)	1 (2.94)		
No	7 (63.63)	4 (36.36)		

*Liver cirrhosis*			3.995	0.046
Yes	5 (62.50)	3 (37.50)		
No	35 (94.59)	2 (5.41)		

*Intrahepatic spread*			6.544	0.011
Yes	3 (50.0)	3 (50.0)		
No	37 (94.87)	2 (5.13)		

*Pathological type*			0.512	0.474
Hepatocellular carcinoma	13 (81.25)	3 (18.75)		
Cholangiocarcinoma	27 (93.10)	2 (6.90)		

*Tumor adjacent to large blood vessels*			5.080	0.024
Yes	4 (57.14)	3 (42.86)		
No	36 (94.74)	2 (5.26)		

**Table 4:** Assignment of factors affecting the therapeutic effect of radiofrequency ablation combined with hepatic artery intervention on patients with recurrence of primary liver cancer after surgery.

Factor	Variable name	Assignment
Tumor diameter	X1	≥3 cm = 1, <3 cm = 0
Envelope intact	X2	No = 1, yes = 0
Liver cirrhosis	X3	No = 1, yes = 0
Intrahepatic spread	X4	No = 1, yes = 0
Tumor adjacent to large blood vessels	X5	No = 1, yes = 0

**Table 5:** Multifactor analysis of the therapeutic effect of radiofrequency ablation combined with hepatic artery intervention on patients with recurrence of primary liver cancer after surgery.

Factor	B	Wald	*P*	OR	95% CI
Tumor diameter ≥3 cm	2.399	6.214	0.009	11.021	8.648 (15.636)
Incomplete envelope	2.515	6.587	0.005	12.367	9.347 (16.647)
Liver cirrhosis	1.936	3.987	0.057	6.932	4.624 (8.346)
Intrahepatic spread	2.277	5.947	0.015	9.746	7.348 (13.314)
Tumor adjacent to large blood vessels	2.098	5.647	0.018	8.148	5.364 (11.365)

## Data Availability

All primary data are available from the corresponding author upon reasonable request.
